# Biliary tract cancers: advances in diagnostic and management

**DOI:** 10.37349/etat.2025.1002328

**Published:** 2025-06-23

**Authors:** James Gutmans, Hiba Mechahougui

**Affiliations:** University of Campania “Luigi Vanvitelli”, Italy; Oncology Department, Geneva University Hospital (HUG), 1205 Geneva, Switzerland

**Keywords:** Biliary tract cancers, cholangiocarcinoma, liquid biopsy, circulating tumor DNA, precision oncology, rare tumors

## Abstract

Biliary tract cancers (BTCs) are aggressive malignancies associated with poor prognosis and limited treatment options. Advances in precision oncology, notably the identification of recurrent molecular alterations such as fibroblast growth factor receptor 2 (*FGFR2*) fusions, isocitrate dehydrogenase 1 (*IDH1*) mutations, *ERBB2* amplifications, and v-Raf murine sarcoma viral oncogene homolog B (*BRAF*) V600E mutations, have introduced new therapeutic avenues and modest survival benefits for patients with advanced disease. However, the practical implementation of targeted therapies remains hampered by challenges in tumor tissue acquisition and molecular testing, highlighting the need for alternative genomic profiling strategies. This comprehensive review examines the role of liquid biopsy as a non-invasive strategy for molecular profiling in BTCs, with a focus on the clinical applications of plasma and bile-derived circulating tumor DNA (ctDNA). We synthesized findings from recent clinical studies evaluating mutation detection rates, concordance between liquid biopsy and tissue-based assays, and the comparative performance of plasma versus bile ctDNA. Liquid biopsy demonstrates high rates of mutation detection and good concordance with tissue analyses. Bile-derived ctDNA, owing to its proximity to the tumor, consistently shows higher sensitivity and mutant allele frequencies (MAFs) than plasma ctDNA. Nevertheless, challenges remain, including lower sensitivity for detecting structural alterations (e.g., gene fusions), variability in ctDNA yield depending on disease status, and a lack of assay standardization across platforms. Liquid biopsy, particularly through bile ctDNA analysis, emerges as a promising adjunct to tissue biopsy for molecular profiling in BTCs. It offers opportunities for earlier, less invasive, and more personalized treatment decisions. Future directions should aim at developing tumor-informed liquid biopsy strategies that increase precision, reduce costs, and ultimately improve patient outcomes. Prospective studies are needed to confirm its clinical utility and survival impact.

## Introduction

Biliary tract cancers (BTCs) are aggressive malignancies with distinct epidemiological and molecular features. It includes intrahepatic cholangiocarcinoma (iCCA), perihilar cholangiocarcinoma (pCCA), distal cholangiocarcinoma (dCCA), and gallbladder cancer (GBC). iCCA originates above the second-order bile ducts, while pCCA and dCCA [collectively called extrahepatic cholangiocarcinoma (eCCA)] are anatomically divided at the cystic duct.

### Incidence and mortality

Globally, pCCA represents the most common subtype, followed by dCCA and iCCA [1]. CCA incidence and mortality vary widely across regions and between subtypes. iCCA rates are highest in Southeast Asia, particularly Thailand, where incidence reaches 85 per 100,000, significantly outpacing Western countries, which report rates below 3.5 per 100,000, making it a rare cancer in this population [2]. Mortality from iCCA has risen globally over the last decade, with sharp increases in Eastern Europe (e.g., Latvia and Lithuania with annual percentage changes exceeding 18%) and moderate rises in North America and Oceania. Conversely, eCCA mortality is generally lower, with only a few countries exceeding 1 per 100,000, such as Hungary and Germany. Trends in eCCA are more variable, with increases in some regions, such as Central Europe, and declines in others, including parts of North America and Australia. These variations reflect differences in risk factors, diagnostic practices, healthcare access, and disease classification [3]. Early-stage diagnosis is rare, with most patients presenting with advanced disease, resulting in a poor 5-year survival rate of 7–20% [1].

### First-line standard therapy: non-molecularly guided approach

The ABC-02 trial [4] established gemcitabine-cisplatin as the first-line standard for unresectable BTCs. Recently, the therapeutic landscape has evolved with the addition of immune checkpoint inhibitors to traditional chemotherapy, offering a new standard of care in first-line treatment [5].

The TOPAZ-1 trial, a phase III study, tested the efficacy of the addition of durvalumab, a programmed cell death ligand 1 (PD-L1) inhibitor to standard chemotherapy in advanced BTCs [5]. Patients with newly diagnosed, inoperable, or metastatic BTCs were randomized to receive either durvalumab or placebo with gemcitabine and cisplatin for up to 8 cycles, followed by durvalumab or placebo maintenance therapy every 28 days until disease progression or withdrawal. Durvalumab significantly improved median overall survival (mOS) (12.9 vs. 11.5 months) and the two-year survival rate was 23.6% for durvalumab versus 11.5% for placebo. These findings represent the most significant advance in first-line BTCs treatment since the ABC-02 trial and led to the Food and Drug Administration (FDA) approval of the durvalumab, gemcitabine, and cisplatin combination in September 2022. The KEYNOTE-966 trial followed TOPAZ-1, evaluating pembrolizumab combined with gemcitabine and cisplatin in treatment-naive metastatic or inoperable BTCs patients [6]. Involving 1,069 patients, this trial showed a median OS of 12.7 months for pembrolizumab versus 10.8 months for placebo. Subgroup analysis revealed that iCCA patients benefited most, compared to eCCA or GBC.

Despite recent therapeutic advances, precision diagnostics remain underexploited in BTCs. This review highlights the emerging role of molecular tools, such as liquid biopsy, in refining patient management, addresses the current gap between this technological innovation and a still limited clinical application, and discusses the persistent technical limitations that must be understood to better inform clinical decision-making.

## Molecular landscape of BTCs and matched therapies

Recent advancements in genetic screening have revealed distinct molecular profiles across CCA subtypes.

iCCA is characterized by frequent mutations in isocitrate dehydrogenase 1 (*IDH1*) (≈ 15–25% [7, 8]) and fibroblast growth factor receptor 2 (*FGFR2*) fusions or rearrangements (≈ 10–45% [7, 8]), which are among the most well-characterized alterations and appear to be mutually exclusive [7]. Less common mutations in iCCA include neurotrophic tyrosine receptor kinase (*NTRK*) gene fusions (4% [9]) and *BRAF* V600E mutations (5% [8]).

pCCAs and dCCAs predominantly harbor *ERBB2* amplifications, *KRAS*, *TP53*, and *SMAD4* mutations, with *KRAS* mutations occurring more frequently than in iCCA [10]. GBC shows amplification of human epidermal growth factor receptor 2 (HER2) in approximately 10–20% of cases [11].

**Figure 1:**
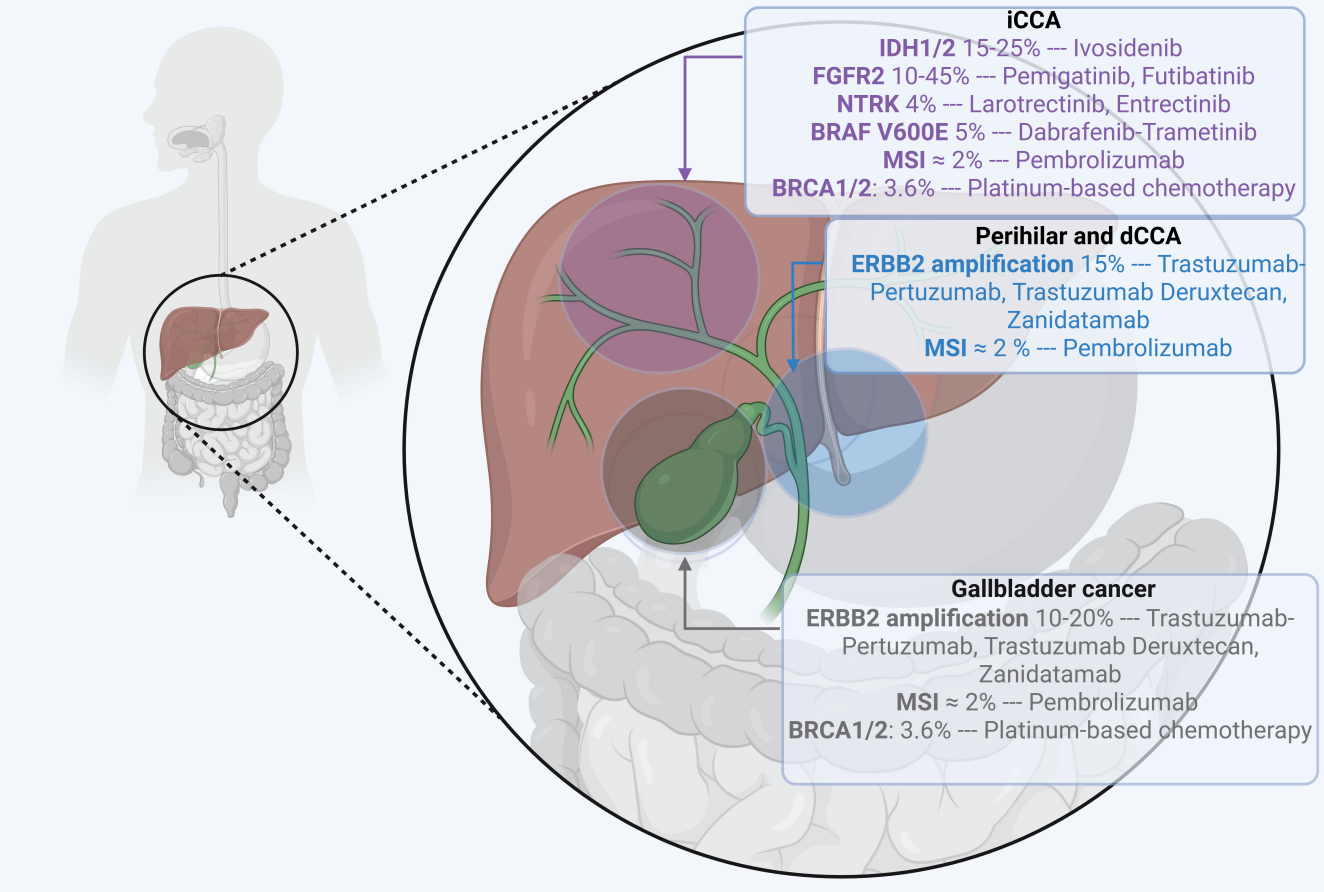
**Molecular alterations in biliary tract cancers and matched targeted therapy.** *BRAF*: v-Raf murine sarcoma viral oncogene homolog B; *BRCA1/2*: breast cancer 1/2; dCCA: distal cholangiocarcinoma; *FGFR2*: fibroblast growth factor receptor 2; iCCA: intrahepatic cholangiocarcinoma; *IDH1/2*: isocitrate dehydrogenase 1/2; MSI: microsatellite instability; *NTRK*: neurotrophic tyrosine receptor kinase. Created in BioRender. Mechahougui, H. (2025) https://BioRender.com/t3pmut8

Although rare (< 2%), DNA mismatch repair deficiency (dMMR) can be observed across all BTCs subtypes [12]. In the TOPAZ-1 trial, dMMR was observed in only 1.5% of cases. Microsatellite instability (MSI) status can be assessed either by immunohistochemistry (IHC) targeting mismatch repair proteins (MLH1, MSH2, MSH6, and PMS2) or by DNA-based assays analyzing microsatellite sequences. The choice between technologies such as next-generation sequencing (NGS), RNA sequencing, or IHC depends on the target alteration and the type of material available, whether tumor tissue or circulating tumor DNA (ctDNA) [11]. Deficiency in any of the four major mismatch repair genes leads to high MSI, a hypermutator phenotype, and increased neoantigen production, making dMMR tumors strong candidates for immune checkpoint blockade [13] (Figure 1).

### *FGFR2* rearrangements

*FGFR* alterations are critical drivers of oncogenesis in CCA and include mutations and rearrangements. FGFR2 functions as a receptor for FGFs and is part of the FGFR1–4 receptor tyrosine kinases family [14]. Under physiological conditions, the FGF/FGFR2 signaling pathway plays important roles in embryonic development, tissue repair, tumor angiogenesis, and proliferation [15] but *FGFR2* fusions or rearrangements can lead to constitutive activation of the receptor, driving tumorigenesis and progression [16]. These alterations are present in approximately 10–15% of patients with iCCA but are almost absent in eCCA and GBC [7]. The identification of *FGFR2* alterations has led to the development of FGFR inhibitors, which have shown clinical benefit in molecularly selected populations.

Pemigatinib, an oral selective and reversible inhibitor of FGFR1–3, demonstrated clinical activity in the phase II FIGHT-202 trial, achieving an objective response rate (ORR) of 35.5% and a mOS of 21.1 months [17]. Based on these results, the U.S. FDA and the European Medicines Agency (EMA) approved pemigatinib as a second-line therapy for advanced BTCs harboring *FGFR2* fusions or rearrangements following progression on systemic treatment [17]. Infigratinib, another selective FGFR1–3 inhibitor, demonstrated an ORR of 23.1% in a phase II trial [18] involving patients with advanced CCA harboring *FGFR2* fusions or rearrangements. The highest response rate (34%) was observed in those who had received only one prior line of therapy. Although approved by the FDA, the application for EMA approval was withdrawn for strategic economic reasons of the sponsor, limiting its availability in Europe [19]. Futibatinib, a highly selective and irreversible FGFR1–4 inhibitor, has demonstrated efficacy even in tumors resistant to other FGFR inhibitors, owing to its irreversible binding. In the phase II FOENIX-CCA1 trial [20], patients with *FGFR2* fusions achieved an ORR of 42% and a mOS of 21.7 months. Futibatinib is now approved by both the FDA and EMA for advanced BTCs with *FGFR2* fusions or rearrangements after progression on systemic therapy. Other FGFR inhibitors, such as lirafugratinib (RLY-4008) [21], derazantinib [22], and erdafitinib [23], have also shown promising early results.

FGFR inhibitors are now being evaluated in first-line settings for iCCA with *FGFR2* fusions. The FIGHT-302 trial (NCT03656536 ) is comparing pemigatinib to gemcitabine-cisplatin in advanced iCCA, while the FOENIX-CCA3 trial (NCT04093362) is evaluating futibatinib in a similar patient population.

### IDH1

*IDH1* encodes an enzyme, IDH1, that plays a critical role in cellular metabolism, DNA transcription, and repair by converting isocitrate to α-ketoglutarate. Mutations in the *IDH1* gene lead to the production of the oncometabolite R-2-hydroxyglutarate (R-2HG), which disrupts epigenetic processes, causes DNA damage, and alters histone methylation, thereby driving tumorigenesis [24].

Ivosidenib, an oral inhibitor of IDH1, was tested in the phase III ClarIDHy trial [25], with an improved mOS (10.3 months with ivosidenib vs. 7.5 months with placebo). After adjusting for crossover, the control group’s mOS was estimated at 5.1 months. Based on these findings, the FDA approved ivosidenib in 2021 for pretreated advanced or metastatic BTCs with proven *IDH1* mutations, and EMA approval followed in 2023. Other IDH inhibitors are under development with CCA cohorts, including olutasidenib [26] and LY3410738 [27]. Resistance mechanisms include secondary mutations, isoform switching, or persistently elevated R-2HG levels [28]. More recently, the tyrosine kinase inhibitor dasatinib is being explored in clinical trials like the phase II trial NCT02428855, that evaluates dasatinib in *IDH*-mutant iCCA.

### *BRAF* V600E

The RAS-RAF-MEK-ERK pathway, frequently activated by *KRAS* mutations across all CCA subtypes, plays a central role in cell proliferation and survival and is linked to poor prognosis [29]. The *BRAF* V600E mutation, a downstream component, is found in 5% of CCA cases [8], predominantly in iCCA, and is associated with advanced disease stages, resistance to chemotherapy, and lower survival rates [30]. The V600E mutation leads to constitutive activation of kinase activity, escaping physiological control and promoting unchecked cell proliferation [31]. These mutations can be detected through NGS, polymerase chain reaction (PCR), or Sanger sequencing, which are superior to IHC analysis for therapy decisions [32].

Due to the rarity of this alteration, the combination of the BRAF inhibitor dabrafenib and the MEK inhibitor trametinib was evaluated in the Rare Oncology Agnostic Research (ROAR) trial, a basket study investigating dabrafenib plus trametinib in *BRAF* V600E-mutated rare cancers [33]. Among 43 BTC patients, the ORR was 47% and the mOS was 14.0 months. Other molecules have been tested, like ulixertinib [34], an ERK 1/2 inhibitor, and selumetinib a MEK inhibitor [35]. Ongoing trials are exploring novel combinations, like PD-L1 inhibitor atezolizumab with the MEK inhibitor cobimetinib (NCT03201458), and the BRAF inhibitor ABM-1310 (NCT05501912 and NCT04190628).

### ERBB2

HER2, encoded by the *ERBB2* gene, is a receptor tyrosine kinase that plays a critical role in tumorigenesis by activating downstream signaling pathways like RAS/MAPK, PI3K/Akt, and JAK/STAT, which contribute to its role in oncogenesis [36]. In eCCA and GBC, *ERBB2* amplification occurs in 10-20% [11].

The phase IIa MyPathway trial [37], a basket study, assessed the combination of pertuzumab and trastuzumab in 39 patients with HER2-positive metastatic BTCs. The ORR was 23%, and the mOS was 10.9 months. Although regulatory approval for BTCs is pending, these results support the potential utility of HER2-targeting monoclonal antibodies. Trastuzumab deruxtecan, an antibody-drug-conjugate (ADC) combining trastuzumab with the topoisomerase I inhibitor deruxtecan, has shown significant efficacy in HER2-positive BTCs in the HERB trial [38]. Patients with HER2-positive BTC (IHC3+ or IHC2+/ISH+) achieved an ORR of 36.4% and a mOS of 7.1 months. Patients with HER2-low BTCs (IHC/ISH < 2+) had lower response rates but still benefited from treatment. The DESTINY-PanTumor02 trial [39] further validated trastuzumab deruxtecan’s efficacy, with an ORR of 56.3% and mOS of 12.4 months in HER2 IHC3+ BTCs. Zanidatamab, a bispecific antibody targeting two HER2 epitopes, has demonstrated rapid and durable responses in HER2-positive BTCs. In the HERIZON-BTC-01 trial [40], patients with *ERBB2*-amplified BTCs achieved an ORR of 41% and a median duration of response of 12.9 months.

Other agents under investigation include TAS0728 (an oral HER2 inhibitor) [41], RC48-ADC [42], and HER2-targeted bispecific antibodies in combination with chemotherapy [43]. While no HER2-targeted therapy is yet FDA or EMA-approved for BTCs, guidelines recommend their use in HER2-expressing cases [11].

### MSI

The KEYNOTE-158 trial [44] evaluated pembrolizumab in MSI-H/dMMR tumors across 27 cancer types, including 22 BTC patients. In this cohort, pembrolizumab achieved a median progression-free survival (mPFS) of 4.2 months, a mOS of 24.3 months, and an ORR of 40.9%. Based on these results, pembrolizumab monotherapy is now recommended in European guidelines and approved by the EMA for advanced BTCs with dMMR or MSI-H after prior systemic therapy [45]. The FDA has also approved pembrolizumab for all MSI-H/dMMR tumors regardless of the cancer type. Ongoing trials, such as the MOST-CIRCUIT trial (NCT04969887), are investigating combination regimens of nivolumab with ipilimumab for MSI-H/dMMR BTCs.

### NTRK

Neurotrophin receptors (TRK A, B, and C) are crucial for cell proliferation and neuronal development [46] through the activation of signaling pathways like PI3K and MAPK [47]. However, *NTRK* alterations, particularly gene fusions, can drive tumorigenesis by causing ligand-independent activation of these pathways [48]. *NTRK* fusions are rare in BTCs, occurring in approximately 4% of cases [9].

Larotrectinib, a first-generation pan-NTRK inhibitor has demonstrated robust efficacy in solid tumors with *NTRK* fusions, including BTCs, in a pooled analysis of 3 clinical trials [49]. In an integrated analysis of three phase I/II trials (ALKA-372-001 [EudraCT 2012-000148-88], STARTRK-1 [NCT02097810], and STARTRK-2 [NCT02568267]), entrectinib, a potent CNS-active TRK inhibitor, showed also durable systemic and intracranial responses in patients with *NTRK*-fusion-positive solid tumors. Entrectinib received FDA approval for use in *NTRK* fusion-positive solid tumors, including CCA, following progression on prior systemic therapy [50]. Both larotrectinib and entrectinib are approved in the United States and Europe for treating unresectable or metastatic solid tumors with *NTRK* gene fusions.

### BRCA

*BRCA* mutations are observed in 3.6% of BTCs, with a higher prevalence of *BRCA2* over *BRCA1* mutations in iCCA and GBC [51]. These mutations are frequently associated with alterations in *TP53*, *ARID1A*, and *KRAS*, among other genes, and are linked to higher rates of MSI and elevated tumor mutational burden, indicating a more immunogenic tumor profile. *BRCA* mutations are associated with improved PFS in patients receiving platinum-based chemotherapy. Furthermore, *BRCA*-mutant tumors exhibit unique genetic and immunogenic characteristics, supporting the rationale for exploring PARP inhibitors in combination with immunotherapy and targeted therapies in this subgroup.

## Diagnostic management and ctDNA

Precise tissue sampling and molecular profiling are critical for the diagnosis and management of CCA. In patients ineligible for curative-intent surgery, core biopsy is recommended to obtain material for histopathological and molecular analyses. However, diagnosis remains challenging due to poor tumor accessibility, particularly in the perihilar region, and biliary cytology achieves a sensitivity of only 20–40% [52]. Differentiating malignant from benign lesions is especially difficult in conditions such as primary sclerosing cholangitis or IgG4-related disease, where inflammatory changes can mimic neoplasia [53]. Misclassification exposes patients to unnecessary major surgeries, with significant associated morbidity and mortality. These limitations have underscored the urgent need for noninvasive diagnostic alternatives. Liquid biopsy, in particular, has gained interest as a complementary tool, especially after procedural restrictions during the COVID-19 pandemic. With tissue biopsy failure rates reaching up to 27% in CCA [54], liquid biopsy now offers a valuable approach for detecting actionable molecular alterations in advanced BTCs.

### ctDNA in localized disease

Cell-free DNA (cfDNA) consists of small DNA fragments, typically 40 to 200 base pairs in length [55] released into the circulation through cellular apoptosis or necrosis. A fraction of cfDNA derived from tumor cells, known as ctDNA, harbors cancer-specific genetic and epigenetic alterations. Detection of ctDNA can be achieved through techniques such as PCR or NGS [56]. While PCR remains cost-effective and suitable for targeted mutation analysis, NGS offers a comprehensive assessment of genomic alterations, an advantage when addressing the genetic heterogeneity characteristic of tumors (Table 1).

**Table 1:** Pros and cons of liquid biopsy in biliary tract cancers

**Pros**	**Cons**
**Minimally invasive, lower risk of complications** Requires only a blood sample, reducing the risk associated with invasive procedures, and the delay.	**Dependency on DNA shedding and tumor burden** Limited efficacy in localized disease.
**Real-time monitoring of secondary mutations** Allows for frequent testing to monitor treatment response and disease progression.	**Technical challenges** Requires highly sensitive and specific assays, which are still under development and standardization.
**Captures tumor heterogeneity** Can detect multiple genetic alterations from different tumor sites, providing a comprehensive genetic profile.	**Limited comprehensive data** Does not provide histological information, which is essential for certain diagnostic and treatment decisions.
**Faster turnaround time** Results can often be obtained more quickly than traditional biopsies, facilitating timely clinical decisions.	**Higher costs and limited availability** Advanced technologies required may be expensive and not widely accessible in all healthcare settings.

Studies in other tumor types, such as non-small cell lung cancer [57], have demonstrated the potential of liquid biopsy in localized settings to assess recurrence risk, refine prognostication, and guide adjuvant chemotherapy decisions. However, in resected CCA, evidence supporting the utility of ctDNA remains limited. A sub-analysis of the phase II STAMP trial [58] evaluated the feasibility of ctDNA to predict recurrence risk during adjuvant therapy in CCA. In this study, ctDNA was analyzed at three time points, before initiation of cisplatin-gemcitabine adjuvant chemotherapy, after five cycles, and after eight cycles, using a tumor-informed assay (Signatera). No significant differences in recurrence-free survival (RFS) or OS were observed based on ctDNA status at these time points. Although patients with detectable ctDNA prior to adjuvant chemotherapy showed a trend toward shorter RFS compared to ctDNA-negative patients, the association did not reach statistical significance. Importantly, patients with persistently positive ctDNA during adjuvant treatment uniformly experienced clinical recurrence, with significantly shorter RFS.

In the curative setting, whether early intervention based on detectable ctDNA, rather than waiting for radiographic recurrence to initiate systemic therapy, can improve outcomes remains unclear. When ctDNA is strongly prognostic for eventual radiographic recurrence but no validated early intervention strategies are available, its detection may not alter management and could instead increase patient anxiety. Isolated case reports [59, 60] have described instances where adjuvant therapy escalation guided by positive ctDNA findings appeared beneficial. Nonetheless, prospective studies specifically designed to determine whether ctDNA-guided interventions translate into meaningful clinical benefits are critically needed.

### First diagnosis and molecular alteration identification in advanced disease

Several retrospective studies have evaluated the utility of ctDNA for initial molecular profiling in advanced BTCs. Mody et al. [61] analyzed 124 patients using a 73-gene ctDNA panel, identifying actionable alterations in 55%, including *FGFR2* fusions, *IDH1/2* mutations, *HER2* amplifications, and *BRAF* mutations. Similarly, Ettrich et al. [62] reported a tissue-blood concordance rate of 74% in therapy-naive CCA patients, rising to 92% in iCCA. Lamarca et al. [63] demonstrated complete concordance between tissue and plasma ctDNA in 112 paired samples from 104 patients, even among those receiving active therapy. Specific targets such as *IDH1* mutations appear particularly well detected by ctDNA, as shown by Aguado et al. [64], who reported a 92% concordance with tissue and observed that clearance of *IDH1* mutations correlated with prolonged PFS in patients treated with ivosidenib. Similarly, Chen et al. [65] detected genetic alterations in 94.8% of ctDNA samples from 154 Chinese patients, with frequencies of *IDH1* mutations and *FGFR2* fusions comparable to tissue results (7.4% vs. 6% and 4.8% vs. 2.7%, respectively). Real-world data support the feasibility of early ctDNA testing. In a 2024 analysis of 1,726 advanced CCA patients [66], actionable alterations were detected in 18% of cases, mainly *IDH1* mutations (11%) and *FGFR2* fusions (9%), with a significant proportion tested before first-line therapy. However, despite these promising findings, sensitivity for detecting structural variants remains suboptimal. Hwang et al. [67] observed an 84.8% sensitivity for ctDNA genomic profiling overall, but only 40% sensitivity for *HER2* amplifications and acknowledged persistent challenges in fusion detection, particularly when ctDNA levels were low.

However, important technical challenges persist, particularly in detecting structural alterations such as *FGFR2* fusions. Berchuck et al. [68], in a large retrospective study of 2,068 ctDNA samples, identified molecular alterations in 84% of patients, with 44% carrying actionable targets. While high concordance rates were reported for *IDH1* (87%) and *BRAF* V600E (100%) mutations, concordance for *FGFR2* fusions was markedly low at 18%. This contrasts sharply with earlier reports from Ettrich et al. [62] and Lamarca et al. [63], suggesting variability in detection likely reflects both biological and technical factors. The Guardant360 assay, which relies on DNA hybrid capture, showed limited sensitivity, particularly for non-*BICC1* fusion partners, due to narrow probe coverage and the challenge of detecting diverse rearrangements in cfDNA [69]. In contrast, assays like Illumina’s TruSight Oncology 500, which specifically targets known *FGFR2* intronic breakpoints, have demonstrated significantly higher detection rates [70]. While cfDNA is a valuable tool for identifying truncal mutations and resistance mechanisms, tissue-based profiling remains essential when fusions are suspected. Advances such as anchored multiplex PCR, broader probe designs [68], and RNA-based methods may improve fusion detection in future practice [71].

In addition to these technical considerations, tumor biology and sampling context can also influence ctDNA accuracy. Okamura et al. [72] found higher concordance between ctDNA and metastatic lesions compared to primary tumors, suggesting that metastatic burden and anatomical site affect detectability.

Beyond detection, prognostic applications of ctDNA have been explored. Yang et al. [73] showed that blood-based copy number variation (CNV) analysis could stratify patients’ immunotherapy responses, with lower CNV risk scores correlating with improved disease control. Likewise, Berchuck et al. [68], showed that higher baseline ctDNA levels were associated with shorter OS, supporting ctDNA as a potential dynamic biomarker of disease burden and outcome (Table 2).

**Table 2:** Selected trials evaluating liquid biopsy in blood in BTCs

**Year**	**Authors**	**Trial type**	**Population**	**Assay**	**Concordance rate liquid/tissue**	**Notable results**
**Localized BTCs**						
2023	Yoo et al. [58]	Randomized phase II	101 patients R0/R1 resected eCCA and regional lymph-node metastases, randomized to gemcitabine-cisplatin versus capecitabine	Signatera, tumor-informed assay	No comparison to tissue	Patients with positive ctDNA before adjuvant chemotherapy had shorter RFS than those with negative ctDNA
**Metastatic BTCs**						
2019	Mody et al. [61]	Retrospective study	124 patients Locally advanced or metastatic BTC (≈ 70% intrahepatic; early-onset < 50 vs. ≥ 50 years)	Guardant^®^	No comparison to tissue	Blood-based liquid biopsy can be used for molecular characterization and can identify clinically relevant alterations including 5% *IDH1* and 7% *FGFR2* mutations
2019	Ettrich et al. [62]	Retrospective study	32 patients Unresectable locally advanced or metastatic cholangiocarcinoma (UICC stage III/IV; gallbladder cancer excluded), all indicated for palliative chemotherapy	QIAamp Circulating Nucleic Acid Kit for ctDNA extraction NGS of 15 gene panel, selected frequently mutated genes	No comparison to tissue	Variant allele frequency correlates with tumor load and PFS 63% of therapy-naive patients experienced changes in their mutational profiles during chemotherapy Patients with mutations via blood-based liquid biopsy in *BAP1*, *PBRM1*, *KRAS*, or *TP53* show a trend toward shorter PFS
2020	Lamarca et al. [63]	Post hoc analysis of patient data collected as part of the prospective ABC-01, -02, and -03	534 patients From the ABC-01/-02/-trial 109 (20.4%) had iCCA; 86 (78.9%) primarily metastatic; 52 (47.7%) with liver-only disease; 66 (60.6%) of these iCCA patients were treated with cisplatin plus gemcitabine	FoundationOne Liquid^®^ Oncomine	*IDH1* mutation: 100% *FGFR2* fusion: 100% *FGFR2* mutation: 100%	High concordance with tissue analysis 40% targetable alterations detected: *IDH1* mutations: 19% *FGFR2* alterations: 10% (5% fusions, 5% mutations) ctDNA before palliative treatment not linked to PFS or OS
2020	Aguado et al. [64]	ctDNA analysis of the randomized phase III trial ClarIDHy	186 patients Previously treated, advanced iCCA	ctDNA/digital PCR	*IDH1*: 92% concordance between plasma ctDNA and tissue samples	mIDH1 detection in plasma ctDNA and tumor tissue was concordant in 92% of samples (193/210) Among ivosidenib-treated patients, *IDH1* mutation clearance occurred in 10/36 (28%) with PFS ≥ 2.7 months versus 0/40 with PFS < 2.7 months No *IDH1* mutation clearance was observed in any placebo-treated patients (*n* = 49), regardless of outcome
2021	Chen et al. [65]	Retrospective study	150 patients Metastatic BTCs	QIAamp Circulating Nucleic Acid Kit for cfDNA extraction	*TP53*: 35.1% in ctDNA vs. 40.4% in tissue samples *KRAS*: 20.1% in ctDNA vs. 22.6% in tissue samples	94.8% of patients showed at least one change detected in their ctDNA Median maximum somatic allele frequency was 6.47% (0.1–34.8%) Higher tumor mutation burden: patients with mutations in *LRP1B*, *TP53*, or *ERBB* family genes had significantly higher tumor mutation burden
2021	Okamura et al. [72]	Observational genomic profiling study conducted under the UCSD-PREDICT prospective protocol (NCT02478931)	121 patients Pathologically confirmed BTCs	Guardant^®^	Overall population: *TP53*: 68% *KRAS*: 80% *PIK3CA*: 90% Metastatic site vs. Primary tumor: *TP53*: 78% vs. 65% *KRAS*: 100% vs. 74% *PIK3CA*: 100% vs. 87%	Common genetic alterations: ctDNA: *TP53* (38%), *KRAS* (28%), *PIK3CA* (14%) Tissue-DNA: *TP53* (44%), *CDKN2A/B* (33%), *KRAS* (29%) Clinical outcomes: Matched therapy: longer PFS (HR 0.60, *P* = 0.047) and higher disease control (61% vs. 35%, *P* = 0.04) Unmatched therapy: shorter PFS and lower disease control
2021	Yang et al. [73]	Multicohort observational analysis	187 patients ICI cohort 1 (*n* = 43): PD-1 inhibitor + lenvatinib ICI cohort 2 (*n* = 108): other ICI-based regimens Non-ICI cohort (*n* = 36): non-ICI therapies	MagMAX cfDNA isolation Kit; TIANamp genomic DNA Kit	No comparison to tissue	CNV detection by liquid biopsy can predict response to immunotherapy Lower CNV risk scores were associated with higher clinical benefit rates in both ICI cohorts Patients with low CNV risk scores exhibited lower rates of PD and higher rates of SD and PR Higher disease control rate was observed in low CNV risk groups compared to high-risk groups Elevated CNV risk scores were linked to increased PD rates in both ICI cohorts
2022	Berchuck et al. [68]	Retrospective, multi-institutional study	1,671 patients Advanced BTCs	Guardant^®^	*IDH1*: 87% concordance between cfDNA and tissue samples *BRAF* V600E: 100% concordance *FGFR2* fusions: 18% concordance	Targetable alterations detected in 44% of patients
2025	Hwang et al. [67]	Retrospective single-center study	102 patients Systemic treatment-naive advanced BTCs (49% iCCA, 26.5% eCCA, 24.5% gallbladder cancer)	Oncomine Comprehensive Assay and AlphaLiquid^®^100 panels	*IDH1* mutations: sensitivity: 100%; PPV: 71.4% *PIK3CA* mutations: sensitivity: 100%; PPV: 83.3% *BRCA1/2* mutations: sensitivity: 100%; PPV: 77.8% *MET* amplifications: sensitivity: 100%; PPV: 100% MSI-high: sensitivity: 100%; PPV: 100% *ERBB2* amplifications: sensitivity: 40.0%; PPV: 100%	ctDNA identified targetable alterations in 34.3% of patients, including *FGFR2* fusions, *IDH1* mutations, MSI, *ERBB2* amplifications, *PIK3CA* mutations, *BRCA1/2* mutations, and *MET* amplification A novel *FGFR2*-*TNS1* fusion was detected via ctDNA analysis The highest ctDNA variant allele frequency is associated with chemotherapy outcome
**Evaluation of resistance mechanisms during treatment**						
2017	Goyal et al. [74]	Prospective translational analysis within the context of the BGJ398 phase II trial	9 patients, 4 of them included in the BGJ398 trial *FGFR2* fusion-positive iCCA	Guardant^®^	No comparison to tissue	All 3 *FGFR2* fusion-positive iCCA patients developed secondary *FGFR2* kinase-domain mutations upon progression 2 patients exhibited multiple distinct *FGFR2* mutations, indicating polyclonal resistance

*BRAF*: v-Raf murine sarcoma viral oncogene homolog B; *BRCA1/2*: breast cancer 1/2; BTCs: biliary tract cancers; cfDNA: cell-free DNA; CNV: copy number variation; ctDNA: circulating tumor DNA; eCCA: extrahepatic cholangiocarcinoma; *FGFR2*: fibroblast growth factor receptor 2; HR: hazard ratio; iCCA: intrahepatic cholangiocarcinoma; ICI: immune checkpoint inhibitor; *IDH1*: isocitrate dehydrogenase 1; MSI: microsatellite instability; NGS: next-generation sequencing; OS: overall survival; PCR: polymerase chain reaction; PD: progressive disease; PD-1: programmed cell death 1; PFS: progression-free survival; PPV: positive predictive value; PR: partial response; RFS: recurrence-free survival; SD: stable disease

### Monitoring therapy and identification of secondary mutations

Early in its development, liquid biopsy was already being explored as a tool for monitoring secondary resistance mutations during targeted therapy. In a phase II trial of *FGFR2*-targeted therapy with BGJ398, Goyal et al. [74] demonstrated the ability of cfDNA to detect acquired resistance alterations, including *FGFR2* V564F and other kinase domain mutations. Similarly, Berchuck et al. [68] identified 31 *FGFR* mutations in plasma ctDNA that were undetectable in the corresponding tumor tissue, including several novel variants of uncertain clinical significance (Table 2).

Follow-up data highlighted the effectiveness of TAS-120, an irreversible pan-FGFR inhibitor, in 4 patients with *FGFR2* fusion-positive CCA who had developed resistance to prior FGFR inhibitors. These patients were selected for TAS-120 treatment based on serial biopsies, ctDNA analysis, and patient-derived tumor cell evaluation [75].

Recently, Goyal et al. [76] gave a new insight on how resistance to FGFR inhibitors emerges in *FGFR2*-altered CCA. Their study, which combined genomic analyses with in vitro and in vivo models, showed that more than 60% of patients who initially respond to treatment later develop secondary *FGFR2* mutations. These include both “gatekeeper” mutations like V565F, which confer high-level resistance through marked impairment of drug binding, and “molecular brake” mutations affecting N550, which are more frequent in clinical samples. Notably, the latter do not substantially reduce inhibitor potency in biochemical or cellular assays, nor do they prevent drug binding. Structural studies suggest that N550 variants induce subtle conformational shifts that allow partial kinase reactivation while preserving some degree of drug interaction, an effect likely amplified in vivo, where FGFR inhibitor concentrations are limited by on-target toxicities and narrow therapeutic windows. These findings support a broader interpretation that resistance in *FGFR2*-altered tumors is shaped less by absolute half-maximal inhibitory concentration (IC50) shifts and more by a dynamic balance between residual signaling activity and pharmacokinetic constraints. Variants like N550K, though only modestly resistant in vitro, may be selectively favored in patients precisely because they retain this balance. This explains the emergence of diverse and often polyclonal resistance patterns, particularly under therapeutic pressure where drug exposure is suboptimal. On this basis, the authors provide a biological rationale for the use of tinengotinib, a multikinase inhibitor with broader target specificity, currently being evaluated in the randomized phase III trial FIRST-308 (NCT05948475).

### Bile-based liquid biopsy

Bile has emerged as a valuable alternative source of ctDNA in BTCs, particularly when tissue sampling is challenging due to biliary obstruction. Given its anatomical proximity to tumor tissue, bile-derived ctDNA offers a promising platform for somatic mutation detection, often surpassing plasma ctDNA in terms of sensitivity and accuracy [77]. For example, Shen et al. [78] investigated bile cfDNA in 6 patients with CCA and 4 with GBC, comparing findings to tumor DNA using a 150-gene panel. Bile cfDNA fragments were found to be longer, closely mirroring the fragment size of tumor DNA, and the assay demonstrated high sensitivity (94.7%) and specificity (99.9%) for detecting single nucleotide variants and indels [78].

Li et al. [79] further emphasized the advantages of bile-derived ctDNA, demonstrating consistently higher concentrations of cfDNA and a greater number of detectable genomic alterations in both bile supernatant and pellet compared to plasma. Mutant allele frequencie (MAF) was also significantly higher in bile samples, with bile-tumor tissue concordance ranging from 85% to 90%. These findings support bile ctDNA as a more reliable representation of tumor-derived genetic material, particularly in tumors with a high mutational burden.

Consistent evidence was provided by Han et al. [80], who reported an 80% concordance between bile ctDNA and tumor biopsy samples in a cohort of 42 BTC patients. Notably, bile ctDNA demonstrated superior sensitivity in detecting mutations in key oncogenes such as *TP53* and *KRAS*, further highlighting its potential for improving molecular diagnostics in BTCs.

Expanding on these observations, Arechederra et al. [81] applied a bile-based NGS panel in 68 patients, achieving a sensitivity of 96.4% and a specificity of 69.2% for malignancy detection. These results collectively underscore the growing role of bile ctDNA as a powerful alternative for genomic profiling, particularly in settings where tissue sampling is limited or inconclusive.

Despite its advantages, logistical challenges, such as the need for bile sampling via endoscopic or surgical procedures, can limit its routine use. However, in cases of biliary obstruction, either at diagnosis or during local recurrence, bile ctDNA presents an attractive option when an endoscopic intervention is required. This approach complements plasma-based liquid biopsy, offering another tool for mutation detection and monitoring in BTCs (Table 3).

**Table 3:** Selected trials evaluating liquid biopsy in bile in BTCs

**Bile cfDNA**						
**Year**	**Authors**	**Trial type**	**Population**	**Assay**	**Concordance rate bile/tissue**	**Notable results**
2019	Shen et al. [78]	Retrospective, single-center observational study	10 patients AJCC stage II-IV BTCs patients (4 gallbladder carcinomas and 6 with CCA)	Customized panel of 150 tumor-related genes	Overall mutation concordance: 90% (9/10) concordant mutations between bile cfDNA and tumor tissue DNA High mutational concordance: 70% (7/10) patients exhibited > 50% mutational concordance between bile cfDNA and tumor tissue DNA	Bile-based liquid biopsy features high concordance with blood samples and tumor tissue: SNV/indel detection: 18 out of 19 tumor variants were detected in bile cfDNA, achieving a sensitivity of 94.7% and specificity of 99.9% CNV detection: 15 out of 20 tumor CNVs were identified in bile cfDNA, with a sensitivity of 75.0% and specificity of 98.9% Therapeutic target CNVs identified in bile cfDNA: *ERBB2*, *CDK6*, *MET*, *PIK3CA*, *ROS1*, *CCNE1*, and *FLT1*
2022	Li et al. [79]	Retrospective single-center study	13 patients AJCC stage I-IV BTCs (7 gallbladder 3 other)	Customized xGen lockdown probe panel with 425 predefined cancer-related genes	Bile vs. plasma vs. tissue: cfDNA yield (*n* = 11): median 1,918 (bile) vs. 63.1 ng/mL (plasma) (*P* = 0.0017) Detection rate: 84.6% vs. 53.8% vs. 100% Median MAF: 1.51–2.68% vs. 1.20% vs. 16.32% Top-10 gene concordance: 90% vs. 85% vs. 35%	Genomic profiling of bile (supernatant/pellet) showed significantly higher concordance with tumor tissue alterations than plasma
2022	Arechederra et al. [81]	Prospective cohort	68 patients Suspicious biliary strictures	Bilemut NGS assay	*KRAS*: 18 mutations were detected in tissue samples, with 5 additional mutations identified exclusively in bile *TP53*: 13 mutations were detected in tissue samples; 4 additional mutations were found only in bile, while 1 mutation present in tissue was not detected in bile	Superior performance demonstrated in 30 paired bile and tissue samples: Sensitivity: 96.4% Specificity: 69.2%

AJCC: American Joint Committee on Cancer; BTCs: biliary tract cancers; CCA: cholangiocarcinoma; cfDNA: cell-free DNA; CNV: copy number variation; MAF: mutant allele frequency; NGS: next-generation sequencing; SNV: single nucleotide variation

## Conclusions

Targeted therapies have redefined the treatment landscape of BTCs, and as they expand into the first-line setting, ctDNA is emerging as a central tool in guiding therapeutic decisions. In real-world practice, early ctDNA testing can identify actionable alterations such as *IDH1* mutations or *FGFR2* fusions, facilitating timely initiation of matched treatments. This non-invasive approach is particularly valuable when tissue access is limited or biopsy material is insufficient, a common challenge in BTCs.

Beyond baseline profiling, serial ctDNA analysis offers a means to monitor tumor evolution and detect emerging resistance mechanisms. In other malignancies, such as EGFR T790M in non-small cell lung cancer or c-KIT secondary mutations in gastrointestinal stromal tumors, this has already translated into routine clinical practice. In BTCs, acquired mutations affecting the FGFR2 kinase domain have been identified through cfDNA analysis and are increasingly recognized as markers of therapeutic resistance. While the adaptation of treatment based on such resistance mutations is not yet standard care in BTCs, some next-generation FGFR inhibitors designed to overcome these alterations are currently under investigation [82]. Their clinical integration may soon enable a more dynamic, mutation-guided sequencing of therapies.

Nevertheless, analytical and biological limitations persist. Sensitivity for detecting amplifications and structural variants varies considerably across platforms; PCR-based assays may better detect predefined alterations, while NGS panels offer broader coverage but may struggle with complex rearrangements such as *FGFR2* fusions, especially when involving rare partners [68]. RNA-based liquid biopsy approaches and anchored multiplex PCR are promising developments that may improve fusion detection. Moreover, ctDNA yield and interpretability depend on tumor biology, with metastatic lesions generally shedding more detectable DNA than primary or low-volume disease. This variability must be accounted for in clinical interpretation.

Looking ahead, ctDNA analysis may extend its utility beyond advanced disease. Several prospective trials, such as NCT05743959, NCT06171321, NCT04183712, and NCT06416397, are evaluating its role in detecting minimal residual disease (MRD) and anticipating recurrence in the adjuvant and surveillance settings. These applications are aligned with approaches being developed in colorectal and lung cancers, where MRD-guided interventions are under clinical validation.

To fully realize its potential in BTCs, ctDNA testing must be integrated into a framework that is both biologically informed and clinically actionable. The development of tumor-informed, stage-adapted, and alteration-specific strategies, coupled with prospective validation and standardized platforms, will be essential to move from technical feasibility to routine clinical impact.

## Abbreviations

ADC: antibody-drug-conjugate
*BRAF*: v-Raf murine sarcoma viral oncogene homolog B
BTCs: biliary tract cancers
CCA: cholangiocarcinoma
cfDNA: cell-free DNA
CNV: copy number variation
ctDNA: circulating tumor DNA
dCCA: distal cholangiocarcinoma
dMMR: DNA mismatch repair deficiency
eCCA: extrahepatic cholangiocarcinoma
EMA: European Medicines Agency
FDA: Food and Drug Administration
*FGFR2*: fibroblast growth factor receptor 2
FGFs: fibroblast growth factors
GBC: gallbladder cancer
HER2: human epidermal growth factor receptor 2
iCCA: intrahepatic cholangiocarcinoma
*IDH1*: isocitrate dehydrogenase 1
IHC: immunohistochemistry
mOS: median overall survival
MRD: minimal residual disease
MSI: microsatellite instability
NGS: next-generation sequencing
*NTRK*: neurotrophic tyrosine receptor kinase
ORR: objective response rate
OS: overall survival
pCCA: perihilar cholangiocarcinoma
PCR: polymerase chain reaction
PD-L1: programmed cell death ligand 1
PFS: progression-free survival
R-2HG: R-2-hydroxyglutarate
RFS: recurrence-free survival

## Declarations

### Copyright

© The Author(s) 2025.
